# Associations between Family Weight-Based Teasing, Eating Pathology, and Psychosocial Functioning among Adolescent Military Dependents

**DOI:** 10.3390/ijerph17010024

**Published:** 2019-12-18

**Authors:** Arielle T. Pearlman, Natasha A. Schvey, M. K. Higgins Neyland, Senait Solomon, Kathrin Hennigan, Rachel Schindler, William Leu, Dakota Gillmore, Lisa M. Shank, Jason M. Lavender, Natasha L. Burke, Denise E. Wilfley, Tracy Sbrocco, Mark Stephens, Sarah Jorgensen, David Klein, Jeffrey Quinlan, Marian Tanofsky-Kraff

**Affiliations:** 1 Department of Medical & Clinical Psychology, Uniformed Services University of the Health Sciences, Bethesda, MD 20814, USA; arielle.pearlman.ctr@usuhs.edu (A.T.P.); mary.neyland.ctr@usuhs.edu (M.K.H.N.); senait.solomon.ctr@usuhs.edu (S.S.); kathrin.hennigan.ctr@usuhs.edu (K.H.); rachel.schindler.ctr@usuhs.edu (R.S.); william.leu@usuhs.edu (W.L.); dakota.gillmore@usuhs.edu (D.G.); jason.lavender.ctr@usuhs.edu (J.M.L.); tracy.sbrocco@usuhs.edu (T.S.); marian.tanofsky-kraff@usuhs.edu (M.T.-K.); 2 Department of Family Medicine, Uniformed Services University of the Health Sciences, Bethesda, MD 20814, USA; david.a.klein26.mil@mail.mil (D.K.); jeffrey.quinlan@usuhs.edu (J.Q.); 3 Military Cardiovascular Outcomes Research Program (MiCOR), Bethesda, MD 20814, USA; lisa.shank.ctr@usuhs.edu; 4 Metis Foundation, 300 Convent St #1330, San Antonio, TX 78205, USA; 5 Department of Psychology, Fordham University, Bronx, NY 10458, USA; nburke12@fordham.edu; 6 Department of Psychological and Brain Sciences, Washington University in St. Louis, St. Louis, MO 63110, USA; wilfleyd@wustl.edu; 7 Department of Family and Community Medicine, Pennsylvania State University, Old Main, State College, PA 16801, USA; mstephens3@pennstatehealth.psu.edu; 8 Fort Belvoir Community Hospital, Fort Belvoir, VA 22060, USA; sarah.k.jorgensen.civ@mail.mil; 9 Malcolm Grow Medical Clinics and Surgery Center, Joint Base Andrews, MD 20762, USA

**Keywords:** weight-based teasing, adolescents, military dependents, eating pathology, obesity

## Abstract

Weight-based teasing (WBT) by family members is commonly reported among youth and is associated with eating and mood-related psychopathology. Military dependents may be particularly vulnerable to family WBT and its sequelae due to factors associated with their parents’ careers, such as weight and fitness standards and an emphasis on maintaining one’s military appearance; however, no studies to date have examined family WBT and its associations within this population. Therefore, adolescent military dependents at-risk for adult obesity and binge-eating disorder were studied prior to entry in a weight gain prevention trial. Youth completed items from the Weight-Based Victimization Scale (to assess WBT by parents and/or siblings) and measures of psychosocial functioning, including the Beck Depression Inventory-II, The Rosenberg Self-Esteem Scale, and the Social Adjustment Scale. Eating pathology was assessed via the Eating Disorder Examination interview, and height and fasting weight were measured to calculate BMI*z*. Analyses of covariance, adjusting for relevant covariates including BMI*z*, were conducted to assess relationships between family WBT, eating pathology, and psychosocial functioning. Participants were 128 adolescent military dependents (mean age: 14.35 years old, 54% female, 42% non-Hispanic White, mean BMI*z*: 1.95). Nearly half the sample (47.7%) reported family WBT. Adjusting for covariates, including BMI*z*, family WBT was associated with greater eating pathology, poorer social functioning and self-esteem, and more depressive symptoms (*ps* ≤ 0.02). Among military dependents with overweight and obesity, family WBT is prevalent and may be linked with eating pathology and impaired psychosocial functioning; prospective research is needed to elucidate the temporal nature of these associations.

## 1. Introduction

Family dynamics and functioning are critical in shaping a child’s identity and self-concept. Further, specific parenting styles are associated with numerous facets of child development and adjustment. For instance, an authoritative parenting style, characterized by both high responsiveness and high demands, is generally associated with optimal functioning among children and adolescents, including better school performance [1,2], higher self-esteem [3] and increased self-regulation [4]. In another study comparing four parenting styles across four countries, an indulgent parenting style, characterized by high warmth and low strictness, was most robustly associated with multiple facets of the adolescent’s self-esteem, including academic, social, emotional, and physical (e.g., “I am an attractive person”) [5], indicating that parental warmth and flexibility is linked with greater child self-acceptance and appearance satisfaction. In contrast, both neglectful and authoritarian (low responsiveness and warmth, and high demands) parenting styles are linked to poorer child outcomes, including low self-esteem, poor school adjustment and antisocial behavior [6]. In addition to parenting styles, family cohesion more broadly may be a protective factor for youth and may bolster resilience [3]. Given data indicating that the family environment is a critical determinant of adolescent adjustment, it is important to elucidate specific familial factors that might confer additional risk for low self-esteem and poor psychosocial functioning. One such aspect of family functioning that warrants additional study is the experience of weight-based teasing (WBT) perpetrated by parents and siblings.

Weight-based teasing is frequently reported by youth with high weight [7,8], and may take many forms, including name-calling, physical bullying, and relational victimization and exclusion [9]. In fact, among adolescents, high weight status is the most commonly reported reason for bullying in schools [10]. Further, WBT may be perpetrated by various sources, including parents, siblings, peers, teachers, and medical professionals [11]. While youth of all weight strata are vulnerable to WBT, evidence indicates that higher body mass index (BMI) is associated with a greater likelihood of verbal, physical and relational peer victimization [9] and that youths with obesity are more likely to report overt WBT as compared to youths with overweight [12,13].

A substantial body of literature has linked WBT with a host of negative psychological and behavioral correlates and consequences. These include unhealthy eating and weight-control behaviors [14,15,16,17,18,19], such as binge-eating and fasting, avoidance of physical activity [15,18,20,21,22], lower self-esteem [23], depression, anxiety, suicidality, body dissatisfaction [17,23,24,25,26,27], internalizing negative weight-based stereotypes [14], and poorer school performance [28]. Longitudinal research has also demonstrated that the experience of WBT in youth confers risk for additional weight and fat gain over time, even after adjusting for baseline values of these characteristics [7,19,29]. Thus, WBT may adversely affect a child’s psychosocial functioning and academic achievement, while also promoting inappropriate weight gain and eating pathology through a number of mechanisms.

Preliminary evidence shows that the nature and effects of WBT may differ depending on the source of teasing. For instance, in one study, the association between WBT in adolescence and higher BMI and obesity 15 years later was present among women for both peer- and family-teasing, whereas for men the association was only observed for peer-based teasing [19]. Additionally, evidence shows that parental criticism of a child’s weight is associated with poorer well-being and unhealthy eating behaviors [30]. Another study found that among children ages 9–12, the most frequent perpetrators of negative weight-based comments were siblings. Further, siblings were more likely to verbally tease, whereas mothers were more likely to express health concerns due to the child’s weight. Of note, parental weight-related comments, even if well-intended, may still be perceived as stigmatizing or shaming to the child [31]. Data also show that adolescents with overweight and obesity report greater pressure to be thin from mothers, fathers, and same sex friends than their lean peers. Pressure to be thin among these youths was associated with impaired insulin action as well as both BMI and fat gain one-year later [29,32].

Various studies highlight the prevalence of WBT from family members [9,33,34,35]. For example, one study among adolescents of all weight strata found that 29% of girls and 16% of boys reported weight-related teasing from a family member [23]. Other studies note that parents provide less financial support for college for their children with overweight or obesity compared to children with average weight [36,37]. In addition, weight stigmatization by family members often continues into adulthood. A study of over 2000 women (mean age: 50 years) with overweight or obesity found that 72% of participants rated family members as the most frequent source of weight stigma, often in the form of teasing, name calling, and inappropriate weight and shape-related comments [38].

Given the pervasiveness and significant psychosocial consequences of WBT among youth, data are needed to elucidate subgroups that may be particularly at high-risk for both WBT and psychosocial difficulties. One such subgroup may be the children of military service members. Service members are subject to weight and fitness standards that may promote weight-based stigma [39]. These standards may perpetuate the notion that individuals are responsible for their weight status and that an inability to maintain a lean physique is the result of lack of willpower or self-discipline. Given the strong cultural emphasis on leanness and fitness endemic to the military, it is possible that service members and their family members internalize weight-biased attitudes and beliefs, and may, in turn, stigmatize or tease their family members with high weight. Given that the prevalence of overweight and obesity in the military has tripled in the past 15 years [40,41], with estimates that up to 60% of active duty military members may be classified as having overweight/obesity and that the children of individuals with high weight are more likely to have overweight and obesity [42], the children of service members may be especially vulnerable to both high weight and WBT within the home.

Further putting these youth at risk, military dependents often face unique stressors specific to their parents’ careers. For instance, parental deployments, relocations, and school transfers [43] are common. Military families move, on average, every two to three years, meaning that a child in a military family may move up to six times within their school career. As a result, military dependents may lack the continuity in their community, school, and friend groups that civilian children possess; therefore, the family unit may be a particularly important source of support and consistency. Thus, it is possible that teasing from family members may be especially salient and aversive to military dependents.

Importantly, the children of service members appear to be at greater risk for emotional difficulties, as well as eating-and mood-related psychopathology as compared to their civilian peers [44,45,46,47]. For example, the absence of a parent during deployment is associated with poorer school performance and elevated internalizing and externalizing symptoms [45,46,48]. Parental deployments also interact with parental distress, such that among those with high parental distress, parental deployments are associated with greater disordered eating attitudes and behaviors among adolescent children [49]. Taken together, data indicate that these youth may be highly vulnerable to both WBT and mood and eating-related disturbances. However, adolescent military dependents are an understudied group and no research, to date, has examined family WBT and its associations with eating pathology and psychosocial functioning among these youths. Elucidation of specific factors that might increase risk for emotional and eating-related disturbances is critical for early intervention to ensure the health and welfare of this important population. Therefore, we examined WBT by immediate family members (parents and siblings) in relation to disordered eating attitudes and psychosocial indices among adolescent military dependents at high-risk for adult obesity and eating disorders. It was hypothesized that family WBT would be associated with greater body dissatisfaction, disordered eating attitudes, and depressive symptoms, and poorer self-esteem and social functioning.

## 2. Materials and Methods

### 2.1. Participants

Adolescent (12–17 years) military dependents were studied at a baseline visit prior to entry in a binge-eating disorder and adult obesity prevention trial (ClinicalTrials.gov ID#: NCT02671292). All were eligible for care in the Military Health System (TRICARE) based on a parent’s current or prior military service, and were identified as high-risk for eating disorders and adult obesity based on: a BMI (kg/m^2^) ≥ 85th percentile [50], indicating the presence of overweight or obesity, and the endorsement of at least one episode of loss-of-control (LOC) eating in the past three months (defined as the subjective feeling of being unable to stop eating, irrespective of the amount of food consumed) [51] and/or elevated anxiety (≥ 32 on the State-Trait Anxiety Inventory for Children) [52]. Recruitment efforts included advertisements online and in base or local periodicals, emails to local listservs, flyers posted at local military hospitals and base facilities, mailings to TRICARE-eligible families, referrals from physicians, and in-person recruitment at study sites. Individuals who expressed interest were screened over the phone to determine initial eligibility.

Exclusion criteria included chronic major medical illness, obesity-related medical complications, major psychiatric disorder (other than binge-eating disorder) that required treatment, weight loss in the last 3 months that exceeded 3% of body weight, or current use or recent discontinuation (within 3 months) of a medication that affected body weight or appetite. Youth taking medications (e.g., selective serotonin reuptake inhibitors, stimulants) were considered for inclusion if their dosage and weight had been stable for 3 months. Girls taking oral contraceptives were eligible if they had been taking the medication for at least 2 months and were weight stable. Individuals currently involved in psychotherapy or a structured weight loss program were not included. Girls were excluded if breastfeeding or if currently or recently pregnant. Participants were compensated for their time and completion of study procedures. This study received approval from the Uniformed Services University Institutional Review Board and the Fort Belvoir Community Hospital (FBCH) Research Office.

### 2.2. Procedures

A baseline screening appointment was completed at military medical facilities within the Greater Washington DC area. Written informed consent for parents and assent for adolescents was obtained prior to initiation of study procedures. Adolescents underwent the following assessments.

#### 2.2.1. Body Mass Index (BMI, kg/m^2^)

Participants’ height (cm) and fasting weight (kg) were measured to calculate BMI. BMI*z*, accounting for age and sex, was determined based on the Centers for Disease Control and Prevention standards of growth [53].

#### 2.2.2. Disordered Eating Attitudes and Behaviors

The Eating Disorder Examination version 12 [54] is a semi-structured interview that was administered by trained members of the research team to assess the presence and frequency of LOC eating episodes in the past three months and disordered eating attitudes and behaviors. The interview generates four subscales (dietary restraint, shape concern, weight concern, eating concern) and a global composite score. The Eating Disorder Examination has shown good reliability and validity among youth [55,56]. In the current sample, Cronbach’s alphas across the four subscales ranged from 0.55 for restraint to 0.85 for shape concern; Cronbach’s alpha for the global score (23 items) was 0.87.

#### 2.2.3. Psychological Functioning

Family WBT was assessed using two items from a Weight-Based Victimization Scale [8,10,24] used in prior studies of adolescents with high BMI. This method is similar to prior studies that have utilized single or two-item measures of family WBT [57]. To assess WBT from family members, participants are asked to indicate whether parents and siblings had “teased or bullied you because of your weight/shape in the last year?” Response options ranged from 0 to 4, with 0 indicating Never and 4 indicating Very Often. Sibling and parent teasing were significantly correlated (Pearson’s r = 0.23, *p* = 0.01; Spearman-Brown coefficient = 0.37). Scores were dichotomized such that individuals who answered Rarely, Sometimes, Often, or Very Often to *either* parent or sibling WBT were coded as having experienced family WBT in the past year. Individuals who responded Never to both the parent and sibling items were coded as not having experienced family WBT in the past year.

The State-Trait Anxiety Inventory for Children—A-Trait Scale was used to assess anxiety symptoms [52]. This is a 20-item self-report scale with scores ranging from 20 to 60 (higher scores indicate greater anxiety symptoms). Scores ≥32 indicated elevated anxiety. Sample items include “I worry about making mistakes”, “I worry about things that may happen”, and “Unimportant things run through my mind and bother me”. This scale has shown good reliability and validity among adolescents [58] and demonstrated acceptable reliability in the current sample (Cronbach’s α = 0.77). 

The Beck Depression Inventory-II [59] is a 21-item self-report scale used to assess depressive symptoms over the past 2 weeks. Scores range from 0 to 63, with higher scores indicating greater depressive symptoms. Participants were asked to report on symptoms such as sadness, irritability, agitation, and loss of interest. This measure has demonstrated reliability and validity across both community and clinical samples [60]. The BDI-II demonstrated good reliability in the present sample (Cronbach’s α = 0.85).

The Rosenberg Self-Esteem Scale [61] is a 10-item self-report scale used to measure participants’ self-esteem. Participants were asked to respond to items such as “I feel that I have a number of good qualities” and “On the whole, I am satisfied with myself.” Items are scored on a four-point Likert-scale ranging from Strongly Agree [1] to Strongly Disagree [4]. Scores range from 10 to 40, with higher scores indicating greater self-esteem. This scale has demonstrated good reliability and validity [62] in previous studies as well as the current sample (Cronbach’s α = 0.89).

The Social Adjustment Scale-Self Report [63] is a 23-item scale that assesses overall social difficulties over the past two weeks across the following domains: school, friends, family, and dating. Sample items include “Have you felt lonely or wished for more friends during the last 2 weeks?” and “During the last 2 weeks, have you been feeling that your family let you down or has been unfair to you?” Higher scores indicate more social difficulties. This questionnaire has demonstrated good reliability and validity in prior studies [64]. For the current study, the total score was utilized and demonstrated acceptable reliability (Cronbach’s α = 0.73).

### 2.3. Statistical Analysis

All analyses were performed with SPSS 25.0 (IBM Corp, Armonk, NY, USA). Data were screened for normality and scores on the BDI-II and EDE subscales were log-transformed to improve normality. Extreme but plausible outliers (n = 3), defined a priori as more than three standard deviations from the mean, were recoded to three standard deviations from the mean in order to retain the cases while minimizing their influence [65]. Family WBT was entered into models as a binary independent variable (presence vs. absence). Analyses of covariance, adjusting for age, sex, race (white vs. nonwhite), BMI*z*, elevated anxiety (presence vs. absence), and LOC eating status (presence vs. absence) were conducted to assess differences between youths reporting family WBT and youths not reporting family WBT on measures of eating and mood-related psychopathology and psychosocial functioning. Elevated anxiety and LOC eating status were included as covariates given that the presence of one or both was required for study eligibility. Exploratory analyses of covariance, adjusting for the previously specified variables, were then conducted to assess the associations of parent WBT and sibling WBT separately on dependent variables of interest. All tests were two-tailed and differences were considered significant when *p <* 0.05.

## 3. Results

### 3.1. Participant Characteristics

One-hundred twenty-eight adolescent (12–17 years) military dependents (54% (n = 69) female) with overweight (22.7%) or obesity (77.3%) and LOC eating in the past three months (58.1%) and/or elevated anxiety (93%) were included in the study; 49.2% of the sample reported both LOC in the past three months and elevated anxiety. The average age was 14.35 ± 1.55y (83% of the sample was 15 or younger; 5% were 17 at the time of study initiation); mean BMI*z* was 1.95 ± 0.39. The racial distribution of the sample was 51.6% White, 23.4% Black, 12.5% Multiracial, 3.1% Asian, and 9.4% Other/Unknown; 21.7% identified as Hispanic. Descriptive characteristics can be found in Table 1.

### 3.2. Prevalence of Family Weight-Based Teasing

Responses on the WBT questionnaire were dichotomized to ascertain the proportion of adolescents who reported WBT from parents and siblings. Results indicated that 42.5% of the sample (n = 54) reported WBT from siblings and 21.1% (n = 27) reported WBT from parents; collectively, 47.7% of the sample (n = 61) reported at least one instance of family WBT (siblings and/or parents) in the past year and 15.6% (n = 20) reported WBT from *both* siblings and parents. Family WBT was not significantly associated with BMI*z* [F(1,126) = 1.63, *p* = 0.20], and though a slightly larger proportion of youths with overweight (62.1%) reported family WBT as compared to youths with obesity (43.4%), this difference did not reach significance (χ^2^ = 3.12; *p* = 0.07). The prevalence of family WBT did not differ by participant race, ethnicity, or sex.

### 3.3. Associations of Family Weight-Based Teasing with Eating-Related Psychopathology

**Figure 1:**
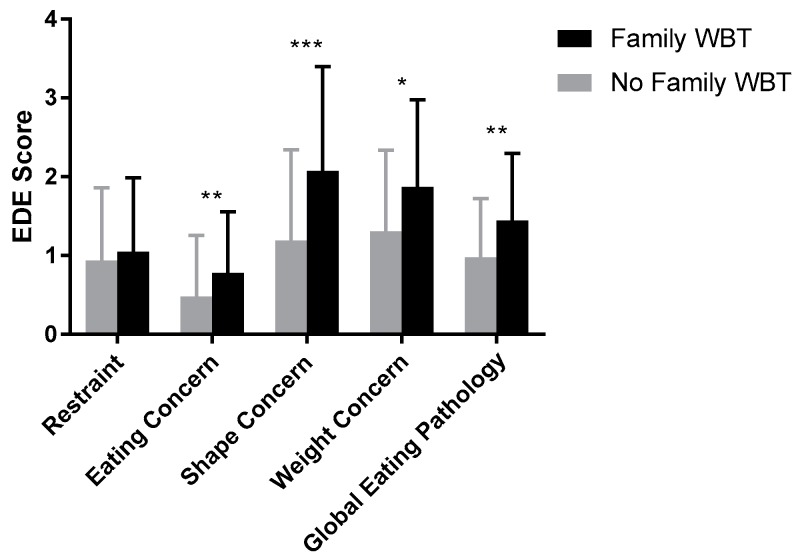
Association of family weight-based teasing with indices of eating pathology. Family weight-based teasing was significantly associated with: Eating Concern (*p* = 0.01), Shape Concern (*p* < 0.001), Weight Concern (*p* = 0.02), and Global Eating Pathology (*p* = 0.01). Adjusted means are shown, controlling for age, sex, race (white vs. nonwhite), BMIz, elevated anxiety (presence vs. absence), and LOC eating status (presence vs. absence). *: *p* < 0.05, **: *p* < 0.01, ***: *p* < 0.001.

Adjusting for age, sex, race, BMI*z*, elevated anxiety status and presence of LOC in the past three months, the presence of family WBT was significantly associated with greater eating concern (F(1,116) = 7.52, *p* = 0.01), shape concern (F(1,116) = 13.97, *p* < 0.001), weight concern (F(1,116) = 5.48, *p* = 0.02), as well as global eating pathology (F(1,116) = 8.24, *p* = 0.01). Only the restraint subscale was not significantly associated with family WBT (*p* = 0.60; see Figure 1).

### 3.4. Associations of Family Weight-Based Teasing with Mood and Psychosocial Functioning

**Figure 2:**
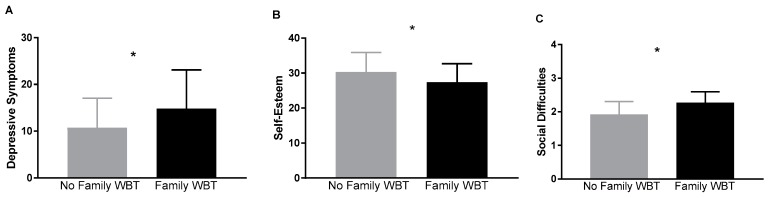
Association of family weight-based teasing with indices of psychosocial functioning. Family weight-based teasing was significantly associated with: Depressive Symptoms ((**A**); *p* = 0.01), Self-Esteem ((**B**); *p* = 0.01), and Social Difficulties ((**C**); *p* = 0.01). Adjusted means are shown, controlling for: age, sex, race (white vs. nonwhite), BMIz, elevated anxiety (presence vs. absence), and LOC eating status (presence vs. absence). *: *p* < 0.05.

After adjusting for previously specified covariates, including BMI*z*, the presence of family WBT was associated with significantly greater depressive symptoms (F(1,116) = 6.93, *p* = 0.01), lower self-esteem (F(1,115) = 6.38, *p* = 0.01), and poorer overall social functioning across domains (F(1,113) = 7.82, *p* = 0.01; See Figure 2).

### 3.5. Exploratory Analyses of Parent- and Sibling-Specific Weight Based Teasing

Parent-specific WBT was significantly associated with lower self-esteem (F(1,115) = 9.81, *p* = 0.002), and social difficulties (F(1,110) = 7.44, *p* = 0.007). The association between parent-specific WBT and greater depressive symptoms approached significance (F(1,116) = 3.85, *p* = 0.05). However, the associations with eating pathology were no longer significant when examining just parent WBT. By contrast, sibling-specific WBT was not associated with social functioning (*p* = 0.19), but was significantly associated with eating pathology (eating concern (F(1,115) = 5.58, *p* = 0.02), shape concern (F(1,115) = 14.96, *p* < 0.001), weight concern (F(1,115) = 5.79, *p* = 0.02), and global score (F(1,115) = 6.58, *p* = 0.01)), depressive symptoms (F(1,115) = 4.28, *p* = 0.04), and poor self-esteem (F(1,115) = 3.58, *p* = 0.03).

## 4. Discussion

The current study assessed the psychosocial correlates of WBT from family members among a sample of adolescent military dependents with overweight and obesity. The findings indicate that teasing from parents and siblings is prevalent among military dependents with overweight and obesity; in fact, nearly half (47.7%) reported at least one instance of WBT perpetrated by siblings or parents. Due to unique stressors faced by military families, such as frequent moves and school changes, immediate family may serve as a primary source of emotional support and continuity among dependents. While family support is critical for adolescents generally [66], it may be even more so for military youth who may lack the consistent school and community support afforded to their civilian counterparts. Therefore, it is possible that WBT from family members may be particularly aversive for adolescent military dependents. Among the current sample, family WBT was associated with multiple facets of eating- and mood-related psychopathology above and beyond the contribution of relevant covariates, including BMI*z*.

Previous studies assessing WBT have varied in the proportion of adolescents reporting WBT from family members. Some studies of youth across weight strata [23,67,68,69] report rates of 20% and 30%, while others [7,26,70] are more consistent with the current findings, reporting that about half of participants report WBT from family members. The current study found that WBT was not correlated with BMI*z* and though a slightly larger proportion of youth with overweight reported WBT compared to those with obesity, this difference was not significant. The lack of a relationship observed between WBT and BMI*z* or weight status is inconsistent with the prior research finding that teasing generally increases with BMI [18,71,72], though this association may be attenuated in samples limited to youth with high body weight [17]. Given that the presence of overweight or obesity was an inclusion criterion in the current study, the lack of association between WBT and BMI may have been due to the constricted range of BMI in our sample.

Family WBT in the present study was associated with greater eating, shape and weight concerns, as well as global eating pathology. This corroborates previous research documenting associations between WBT from family members and body dissatisfaction, disordered eating behaviors, and unhealthy weight control behaviors [23,26,67,68,73]. Furthermore, in line with the previous literature [23,67,68,73], WBT was also associated with depressive symptoms, poorer self-esteem and impaired social functioning. In addition, data demonstrate that greater family dysfunction is linked with exacerbated risk for psychopathology and eating disturbances among youth [74,75]. Specifically, children in families characterized by rigidity, disengagement, and low cohesion may be most susceptible to eating disorders. It is possible that the family separation experienced in military families contributes to reduced cohesion and intermittent periods of relative disengagement, which may then exacerbate risk for poor outcomes in children. Therefore, WBT from family members may interact with perceived family functioning to promote eating disorder risk among these youths. Further research is needed to explore these associations.

There are a number of mechanisms that may help to explain the associations observed in the present study. More specifically, it is plausible that the experience of WBT induces feelings of body dissatisfaction and negative affect [8,22,24], which collectively place an adolescent at-risk for undue concern with shape and weight and aberrant eating behaviors. Whereas some have proposed that weight stigma might motivate weight loss behaviors [76], the current study found no association between family WBT and dietary restraint. Thus, within the current sample, WBT was *not* associated with attempted dieting or caloric restriction, but *was* associated with both shape and weight dissatisfaction. Therefore, the current findings provide further evidence that WBT from parents and siblings is not a useful tool to motivate healthy behaviors and that, in contrast, it may actually promote disordered eating and body dissatisfaction which may ultimately place an adolescent at-risk for eating disorders and low self-esteem. However, given the relatively low reliability of the restraint subscale observed in the current study, as well as others [77], the lack of a significant association with WBT should be interpreted with caution.

The observed associations may be contextualized within a social identity threat framework [73]. Specifically, experiences of WBT and stigma may threaten the social identity of individuals with overweight or obesity, resulting in both acute and chronic stress which, in turn, reduces one’s self-regulatory capacity and increases one’s desire to escape from or avoid the stigmatized identity [78]. In this instance, reduced self-regulatory capacities may promote overeating or disinhibited eating behaviors. Further, poor self-regulation might also place youth at risk for greater family discord and conflict. Efforts to avoid and escape stigma may lead to body dissatisfaction, unhealthy weight control behaviors, and reduced participation in social, academic, and family settings. Lastly, using food to cope is a well-documented response to stress [79,80] and weight stigma [9,38]. Collectively, the stress resulting from WBT may negatively impact mental and physical health; additional research is needed to elucidate components of the social identity threat framework among this population.

Poor self-esteem in adolescence may negatively affect social engagement, school participation, and academic self-concept, thereby cumulatively affecting psychosocial functioning across domains. This is supported by previous research demonstrating reciprocal relationships between these constructs [81]. It may also be that youth experiencing WBT at home have greater depressive symptoms that may make them more reticent to engage in social groups, attend school, or participate in class discussions, thereby adversely affecting both social and school functioning.

While the results from analyses assessing sibling- and parent-teasing separately are preliminary, it seems that both parent and sibling WBT are associated with negative correlates. However, sibling WBT may be associated more robustly with eating pathology, whereas parent WBT may be more closely linked to poor social functioning and negative self-evaluation. This may be due to the different forms of WBT perpetrated by siblings (e.g., verbal teasing) versus parents (e.g., pressure to lose weight) observed in prior research [31]. Additional research is needed to further elucidate the effects of different sources and forms of teasing.

Strengths of the current study include that the assessment of an understudied, vulnerable, and hard-to-reach group. Moreover, military dependents are a group for whom family WBT may be both particularly prevalent and distressing. In addition, the current study utilized a well-validated semi-structured interview assessment of eating-related pathology and measured height and weight from which BMI*z* was determined. Limitations include the narrow eligibility criteria of the study. Specifically, all youth were seeking prevention of adult obesity and eating disorders and were required to have an elevated BMI percentile (≥85th) and to report the presence of elevated anxiety symptoms and/or the presence of LOC eating. Thus, the results may not be generalizable to the broader adolescent military dependent population or to military dependents who are not at-risk for adult obesity and eating disorders. Given that all youths in the study were at-risk for both conditions, the current sample may represent a specific subset of military dependent youths who are particularly vulnerable to both eating- and mood-related pathology and the presence of WBT, independently. However, significant associations between WBT and psychosocial impairments were found even after adjusting for BMI*z* and LOC eating status, thereby indicating that WBT is uniquely associated with psychosocial impairments among these youths. Future research should assess these constructs among non-intervention-seeking samples of military dependent adolescents of a broader weight spectrum.

Our study was also limited by the lack of a matched civilian control group; thus, we are unable to identify whether the prevalence and correlates of family WBT differ among military dependents. Further, the cross-sectional study design limits our ability to draw any causal conclusions, therefore, it may be possible that youth with greater psychosocial impairments are more conscious of and sensitive to weight-related comments and therefore, are more likely to report WBT. It may also be the case that an unmeasured third variable better accounts for the association between family WBT and eating and mood-related distress. For instance, youths with poor relationships with their parents and/or siblings may be both more likely to perceive WBT from these individuals and to report low mood and psychosocial disturbances as a result of these problematic relationships. Nonetheless, the current study is an important first step in elucidating the prevalence of family WBT among adolescent military dependents and its associations with eating pathology and psychosocial functioning.

Future research should utilize a matched civilian comparison group to ascertain whether WBT is more prevalent or more robustly associated with mood and eating-related pathology among military dependent youth as compared to civilians. It will also be relevant to determine whether there are additive effects of WBT among this population; for instance, future adequately powered research should assess whether youths reporting WBT from both parents *and* siblings fare worse than youths reporting WBT from one source, but not the other. Additionally, studies should elucidate the specific types of family WBT experienced by military dependents. As prior studies indicate that WBT may take different forms [11,82], additional research is needed to parse whether different forms of teasing are associated with specific sequelae; for instance, it may be the case that stated health concerns, which are more likely to come from parents, affect children differently than overt teasing or name-calling, which are more likely to be perpetrated by siblings. Of note, previous research has indicated that even subtle pressure to be thin (such as receiving compliments due to weight loss) from family members is linked with adverse health correlates [32] and outcomes [29]; thus, it may be that weight-related comments generally, irrespective of the nature and intended purpose, are experienced as distressing to youth. This is supported by previous research showing that even parent weight talk (e.g., comments about one’s own weight) is associated with greater depressive symptoms and unhealthy weight control methods [83] among children.

If the current results are supported by prospective studies, efforts may be warranted to educate the family members of adolescent military dependents with high weight about the potentially harmful sequelae of WBT within the home. Given the proportion of youths reporting family WBT in the current study, it may also be beneficial for providers working with adolescent military dependents with high weight to assess WBT within the home and to encourage discussions with family members about weight-related comments that are experienced as distressing. It is possible that parents and siblings are not aware of the distress associated with their weight-based comments and teasing; therefore, it may be important to encourage youth to communicate their feelings about and reactions to weight-related comments openly with family members.

## 5. Conclusions

The current study assessed the prevalence of weight-based teasing from parents and siblings among a sample of adolescent military dependents at high-risk for adult obesity and binge-eating disorder. The findings indicated that nearly half of respondents reported the presence of weight-based teasing from their parents and/or siblings and that this form of teasing was significantly associated with eating pathology and poorer psychosocial functioning, above and beyond the contribution of demographics and BMIz. Exploratory analyses revealed that while both parent- and sibling-WBT were independently associated with adverse psychosocial correlates, the pattern of these associations differed by source. Should these results be confirmed in more heterogeneous samples of military dependents and supported by prospective data, studies to address and reduce WBT within the military family may be warranted.

## Figures and Tables

**Table 1:** Baseline Participant Demographics by Teasing Status.

	Family WBT: Presence (n = 61)			Family WBT: Absence (n = 67)				Total Sample (n = 128)		
		%	N		%	N	ᵡ^2^		%	N
Sex							1.2			
Female		59	36		49.3	33			53.9	69
Male		41	25		50.7	34			46.1	59
Race							6.7			
Black		19.7	12		26.9	18			23.4	30
White		45.9	28		56.7	38			51.6	66
Asian		4.9	3		1.5	1			3.1	4
Multiple		14.8	9		10.4	7			12.5	16
Other		14.8	9		4.5	3			9.4	12
Ethnicity							0.86			
Hispanic		23	14		17.9	12			20.3	26
Weight Status ^+^							3.1			
With Overweight		29.5	18		16.4	11			22.7	29
With Obesity		70.5	43		83.6	56			77.3	99
Reported Loss of Control in Past Month		54.1	33		41.8	28	1.9		47.7	61
Presence of Elevated Anxiety		93.4	57		92.5	62	0.04		93.0	119
	**M**	**SD**	**N**	**M**	**SD**	**N**	**F**	**M**	**SD**	**N**
Age (y)	14.5	1.6	61	14.2	1.5	67	0.98	14.4	1.5	128
BMI*z*	1.9	0.41	61	1.9	0.37	67	1.6	1.9	0.39	128
EDE Restraint Subscale	1.1	0.97	61	0.94	0.92	67	1.1	1.0	0.95	128
EDE Eating Concern Subscale	0.77	0.71	61	0.46	0.69	67	5.0 *	0.61	0.72	128
EDE Shape Concern Subscale	2.0	1.3	61	1.2	1.2	67	13.8 ***	1.6	1.3	128
EDE Weight Concern Subscale	1.9	1.1	61	1.3	1.0	67	8.8 **	1.6	1.1	128
EDE Global Score	1.4	8.2	61	0.98	0.74	67	10.9 **	1.2	0.81	128
Depressive Symptoms	14.6	8.0	61	10.7	6.3	67	9.4 **	12.6	7.4	128
Self-Esteem	27.6	5.4	60	30.3	5.6	67	7.6 **	29.0	5.6	127
Social Difficulties	2.1	0.39	59	1.9	0.39	66	9.9 **	2.0	0.40	125

^+^ With Overweight: 95th percentile > Body Mass Index ≥ 85th percentile. With Obesity: Body Mass Index ≥ 95th percentile. M: mean; SD: standard deviation. BMI*z*: Age and sex standardized Body Mass Index. EDE: Eating Disorder Examination. Statistical tests conducted: chi-square, one-way analysis of variance. *: *p* < 0.05, **: *p* < 0.01, ***: *p* < 0.001.
